# Optimized gating strategy and supporting flow cytometry data for the determination of the Ki-67 proliferation index in the diagnosis of myelodysplastic syndrome

**DOI:** 10.1016/j.dib.2022.107976

**Published:** 2022-02-22

**Authors:** Stefan G.C. Mestrum, Eline M.P. Cremers, Norbert C.J. de Wit, Roosmarie J.M. Drent, Frans C.S. Ramaekers, Anton H.N. Hopman, Math P.G. Leers

**Affiliations:** a Department of Molecular Cell Biology, GROW-School for Oncology and Developmental Biology, Maastricht University Medical Center, Maastricht, the Netherlands; b Department of Clinical Chemistry and Hematology, Zuyderland Medical Center, Sittard-Geleen, the Netherlands; c Department of Internal Medicine, Division of Hematology, Maastricht University Medical Center, Maastricht, the Netherlands; d Department of Hematology, Radboud University Medical Center, Nijmegen, the Netherlands; e Central Diagnostic Laboratory (CDL), Maastricht University Medical Center, Maastricht, the Netherlands; f Nordic-MUbio, Susteren, the Netherlands

**Keywords:** Flow cytometry, Data, Gating procedure, Proliferation, MDS, Ogata score, Ki-67

## Abstract

This Data in Brief article presents a novel flow cytometric assay used to acquire and process the data presented and discussed in the research paper by Mestrum et al., co-submitted to *Leukemia Research,* entitled: “Integration of the Ki-67 proliferation index into the Ogata score improves its diagnostic sensitivity for low-grade myelodysplastic syndromes.” [1]. The dataset includes the gated fractions of the different myeloid populations in bone marrow (BM) aspirates (total BM cells, CD34 positive blast cells, erythroid cells, granulocytes and monocytes. The raw data is hosted in FlowRepository, while the analyzed data of 1) the fractions of the different myeloid cell populations and 2) the Ki-67 proliferation indices of these myeloid cell populations are provided in tabular form to allow comparison and reproduction of the data when such analyses are performed in a different setting. BM cells from aspirates of 50 myelodysplastic syndrome (MDS) patients and 20 non-clonal cytopenic controls were stained using specific antibody panels and proper fixation and permeabilization to determine the Ki-67 proliferation indices of the different myeloid cell populations. Data was acquired with the three laser, 10-color Navios™ Flow cytometer (Beckman Coulter, Marseille, France) with a blue diode Argon laser (488 nm, 22 mW), red diode Helium/Neon laser (638 nm, 25 mW) and violet air-cooled solid-state diode laser laser (405 nm, 50 mW). A minimum of 100,000 relevant events were acquired per sample, while we aimed at acquiring 500,000 events per sample. Gating was performed with the Infinicyt v2.0 software package (Cytognos SL, Salamanca, Spain). These data may guide the development and standardization of the flow cytometric analysis of the Ki-67 proliferation index (and other markers for cell behavior) for differentiation between non-clonal cytopenic patients and MDS patients. In addition, this assay may be used in myeloid malignancies for research and clinical purposes in other laboratories. This data can be used to encourage future research regarding stem-/progenitor cell resistance against anti-cancer therapies for myeloid malignancies, diagnostics of myeloid malignancies and prognosis of myeloid malignancies. Therefore, these data are of relevance to internist-hematologists, clinical chemists with sub-specialization of hematology and hemato-oncology oriented researchers.

## Specifications Table

**Table:** 

Subject	Oncology
Specific subject area	Flow cytometric analysis of the Ki-67 proliferation index in non-clonal cytopenic and myelodysplastic syndrome (MDS) patients.
Type of data	Table Figure Database (XLS)
How the data were acquired	Navios™ Flow cytometer (Beckman Coulter, Marseille, France). Three laser, 10-color flow cytometer: blue diode Argon laser (488 nm, 22 mW), red diode Helium/Neon laser (638 nm, 25 mW) and violet air-cooled solid-state diode laser laser (405 nm, 50 mW). FITC fluorescence: 550 nm dichroic filter; 525/40 nm band pass (BP) filter. PE fluorescence: 595 nm dichroic filter; 575/30 nm BP filter. ECD fluorescence: 655 nm dichroic filter; 620/30 nm BP filter. PE-Cy5.5 fluorescence: 730 nm dichroic filter; 695/30 nm BP filter. PE-Cy7 fluorescence: 755 nm long pass (LP) filter, APC: fluorescence 710 nm dichroic filter; 660/20 nm BP filter. APC-A700 fluorescence: 750 dichroic filter; 725/20 nm BP filter. APC-A750 fluorescence: 755 nm LP filter, PB fluorescence: 480 nm dichroic filter; 450/40 nm BP filter. KO fluorescence: 550/40 nm BP filter.
	Instrument setup was performed according to standard procedures. Data collection was performed with the Navios™ Flow Cytometer in combination with the Navios Tetra software (Beckman Coulter, Marseille, France). Verification of the optical alignment and fluidics system of the Navios™ Flow Cytometer was performed using Flow-Check™ Pro Fluorospheres (Beckman Coulter). The verification of the compensation for each fluorochrome was established using Flow-Set™ Pro Fluorospheres (Beckman Coulter) and was performed weekly. A minimum of 100,000 relevant events were acquired per sample, while we aimed at acquiring 500,000 events per sample. We ensured that at least 100 Ki-67 positive cells per myeloid cell population were measured. Gating was performed with the Infinicyt v2.0 software package (Cytognos SL, Salamanca, Spain).
Data format	Raw Analyzed
Description of data collection	Seventy anemic and/or cytopenic patients that underwent bone marrow (BM) aspiration for routine diagnostic purposes at the Zuyderland Medical Center from 2016 to 2021 were included in this study. This patient group consisted of 20 non-clonal cytopenic controls and 50 patients diagnosed with MDS. Leftover material of the BM aspirates of these patients was used to develop and analyze the Ki-67 immunostaining procedure. Patients with ongoing radio- and/or chemotherapy were not included.
Data source location	• Department of Clinical Chemistry & Hematology, Zuyderland Medical Center • Sittard-Geleen, Limburg • The Netherlands • Latitude: 50.983190; longitude: 5.844600
Data accessibility	Repository name: FlowRepository Data identification number: FR-FCM-Z4VP Direct URL to data: https://flowrepository.org/id/FR-FCM-Z4VP
Related research article	S.G.C. Mestrum, E.M.P. Cremers, N.C.J. de Wit, R.J.M. Drent, F.C.S. Ramaekers, A.H.N. Hopman, M.P.G. Leers. Integration of the Ki-67 proliferation index into the Ogata score improves its diagnostic sensitivity for low-grade myelodysplastic syndromes*,* Leuk Res. 113 (2022) 106789. https://doi.org/10.1016/j.leukres.2022.106789.

## Value of the Data

• These data are useful for development and standardization of the diagnostic flow cytometry protocol including the Ki-67 proliferation index (and other markers for cell behavior) in myeloid malignancies.
• These data are relevant for internist-hematologists, clinical chemists with sub-specialization of hematology and hemato-oncology oriented researchers. The dataset may also be useful for educational purposes.
• These data can be used to develop the flow cytometric assay for determination of the Ki-67 proliferation index in order to differentiate between non-clonal cytopenic patients and MDS patients as published in *Leukemia Research* [1].

## 1 Data Description

Table 1 shows the antibody marker panels that were used to generate the present dataset. By means of these marker panels, the gating strategy shown in Fig. 2 was established to allow gating of the different hematopoietic cell populations of interest. These hematopoietic cell populations included the total BM cells population, CD34 positive blast cells, erythroid cells, granulocytes and monocytes. Gating of these different cell populations generated the fractions that are shown in Tables 2A and 2B. After selecting the different hematopoietic cell populations, the Ki-67 proliferation index was determined with multiple gating approaches (polygonal gating, rectangular gating, predefined thresholds of 40 fluorescent units (FU) and 100 FU. These gating approaches generated the data displayed in the Supplemental Data File.

**Table 1:** Overview of antibody panels used in the present study. Antibodies highlighted in orange represent backbone markers that can be used to merge the different tubes with the Infinicyt 2.0 software.

Panel	FITC	PE	ECD	PC5.5	PE-Cy7	APC	APC-A700	APC-A750	PB	KO
**1**	IgG1			CD13	CD117	CD34			HLA-Dr	CD45
**2**	Ki-67	CD14	CD64	CD13	CD117	CD34	CD10	CD11b	HLA-Dr	CD45

Abbreviations: FITC: fluorescein isothiocyanate; PE: phycoerythrin, ECD: electron-coupled dye; PC5.5: peridinin chlorophyll protein complex 5.5; PE-Cy7: phycoerythrin-cyanine 7; APC: allophycocyanin; APC-A700: allophycocyanin Alexa700; APC-A750: allophycocyanin Alexa750; PB: pacific blue; KO: krome orange.

**Table 2A:** Resulting cell fractions from the gating of the different myeloid cell populations in non-clonal cytopenic control patients.

Non-clonal cytopenic patient	CD34+ blast cells (%)	Erythroid cells (%)	Granulocytes (%)	Monocytes (%)
**1**	0.8	21.0	52.1	3.6
**2**	1.1	51.2	13.3	3.7
**3**	0.5	31.5	14.0	8.4
**4**	0.6	45.7	22.4	5.4
**5**	0.4	47.8	35.2	2.4
**6**	0.2	18.3	70.3	2.0
**7**	0.2	22.6	64.1	1.9
**8**	1.0	13.6	60.6	7.3
**9**	0.9	17.1	59.0	3.8
**10**	0.1	13.2	72.1	2.9
**11**	0.9	34.0	47.5	2.0
**12**	0.4	39.1	16.5	9.7
**13**	0.2	10.6	63.4	3.1
**14**	0.5	7.8	79.3	9.6
**15**	0.2	54.8	33.0	2.4
**16**	0.4	16.5	34.9	2.4
**17**	0.2	36.9	53.2	2.1
**18**	0.3	30.8	56.9	0.7
**19**	0.1	32.2	52.5	3.9
**20**	1.6	33.7	35.0	2.5

**Table 2B:** Resulting cell fractions from the gating of the different myeloid cell populations in MDS patients.

MDS patient	CD34+ blast cells (%)	Erythroid cells (%)	Granulocytes (%)	Monocytes (%)
**1**	1.9	36.0	44.9	5.0
**2**	0.7	35.4	32.7	2.2
**3**	19.9	10.8	2.5	0.4
**4**	3.3	22.0	38.1	4.8
**5**	6.4	16.9	14.0	10.1
**6**	5.1	35.4	24.4	5.4
**7**	6.5	23.1	32.2	4.3
**8**	0.6	24.3	42.7	4.5
**9**	3.9	51.6	18.5	8.6
**10**	0.5	54.9	21.3	5.9
**11**	0.1	30.4	37.7	7.4
**12**	2.7	58.2	22.2	5.6
**13**	9.0	19.6	57.6	1.0
**14**	0.1	53.8	21.2	0.7
**15**	10.3	26.4	40.8	5.4
**16**	7.2	36.6	18.8	1.4
**17**	7.4	22.6	61.1	0.4
**18**	4.8	31.8	19.4	0.2
**19**	0.0	40.3	15.3	9.1
**20**	2.4	29.1	44.0	5.4
**21**	1.6	27.6	54.4	4.0
**22**	0.5	59.0	27.7	1.4
**23**	4.5	34.9	7.1	2.6
**24**	0.5	41.0	39.3	3.7
**25**	0.6	41.1	28.4	3.4
**26**	1.1	60.7	21.0	2.0
**27**	0.7	42.5	29.9	1.9
**28**	0.6	13.7	69.4	3.4
**29**	1.1	17.3	54.6	21.1
**30**	0.9	33.6	87.7	2.3
**31**	0.4	24.8	59.1	2.2
**32**	9.2	45.9	25.2	6.5
**33**	1.0	17.3	60.5	12.9
**34**	0.5	29.3	43.1	8.8
**35**	0.6	28.0	55.4	2.0
**36**	0.1	4.8	87.3	2.0
**37**	0.2	46.7	43.0	0.7
**38**	9.3	24.7	38.1	2.2
**39**	6.4	11.3	60.5	3.0
**40**	2.5	20.4	65.7	3.1
**41**	1.9	21.6	68.5	1.9
**42**	0.9	37.2	42.0	7.3
**43**	1.6	67.8	20.2	0.7
**44**	5.6	49.3	23.6	2.0
**45**	28.7	9.4	26.6	3.7
**46**	2.4	33.7	31.4	19.5
**47**	3.6	55.8	27.1	2.1
**48**	3.4	9.6	61.4	13.7
**49**	0.4	17.2	66.6	2.4
**50**	0.7	48.4	42.1	1.0

Abbreviations: MDS: myelodysplastic syndromes.

The Ki-67 proliferation indices that were determined by the different gating approaches are visualized in Fig. 3. Ki-67 proliferation indices were quantified by manually placed polygonal and rectangular gates. Alternatively, Ki-67 proliferation indices were quantified by placing the gates based on a predefined threshold based on the fluorescent intensity of the Ki-67 staining (40 FU and 100 FU). The highest significance level for the differences between the Ki-67 proliferation index of total BM cells in non-clonal cytopenic patients and that in MDS patients were found by placing rectangular gates (p=0.0047). This difference was not found by placement of polygonal gates (p=0.083) and differences diminished even further by use of predefined gating thresholds based on the fluorescent intensity of the Ki-67 staining (40 FU threshold: p=0.061; 100 FU threshold: p=0.056). For erythroid cells, differences between the Ki-67 proliferation index in non-clonal cytopenic patients and MDS patients were also most apparent by placement of rectangular gates (p=0.015). Gating of the Ki-67 erythroid cells with polygonal gates (p=0.09) and predefined thresholds of 40 FU (p=0.31) and 100 FU (p=0.71) did not result in significant differences between non-clonal cytopenic patients and MDS patients. Rectangular gating produced similar differences as compared to polygonal gating of the Ki-67 proliferation index in CD34 positive blast cells (rectangular gating: p=0.000047; polygonal gating: p=0.0000024) and granulocytes (p=0.0003 and p=0.0008, respectively) in comparisons of the two different patient groups. Differences diminished visually (as can be seen in Fig. 3) by the use of the predefined gating thresholds of 40 FU and 100 FU for gating the Ki-67 proliferation index in CD34 positive blast cells (40 FU: p=0.36; 100 FU: p=0.018) and granulocytes (p=0.0015 and p=0.0001, respectively), but remained significant. For monocytes, none of the gating strategies produces significant differences between the Ki-67 proliferation index in non-clonal cytopenic patients and that in MDS patients (polygonal gating: p=0.95; rectangular gating: p=0.17; 40 FU threshold: p=0.48; 100 FU threshold: p=0.19).

## 2 Experimental Design, Materials and Methods

### 2.1 Sample collection

Seventy patients that were admitted to BM investigation for diagnosis of MDS at the Zuyderland Medical Center from 2016 to 2021 were included in the present dataset. These comprised 20 non-clonal cytopenic control patients and 50 MDS patients. Patients that were undergoing radio- and/or chemotherapy at the time of diagnosis were excluded from the dataset. The routine BM investigation for diagnosis of MDS comprised morphological, cytogenetic and molecular analyses. Meeting the morphological criteria for hematological and/or pathological diagnosis was a prerequisite for the definitive diagnosis of MDS. Leftover material of the routine BM investigation was used to develop and investigate the Ki-67 immunostaining procedure.

### 2.2 Immunocytochemical staining protocols and flow cytometry

Two different antibody panels were used in conjunction with 10 color/12 parameter flow cytometry for determination of the Ki-67 proliferation indices of total BM cells, CD34 positive blast cells, erythroid cells, granulocytes and monocytes [2]. The different antibody panels used in this study are shown in Table 1. Tube 1 was used as an optional negative control and contained 5 µl IgG1 isotype control-FITC (GM-4992, VI-AP, Nordic-MUbio, Susteren, The Netherlands), 5 µl CD13-PE-Cy5.5 (Immu103.44, Beckman Coulter, Marseille, France), 5 µl CD117-PE-Cy7 (104D2D1, Beckman Coulter), 5 µl CD34-APC (581, Beckman Coulter), 5 µl HLA-DR-PB (Immu-357, Beckman Coulter), 2 µl CD45-KO (J.33, Beckman Coulter). Tube 2 comprised a panel of fluorescently labeled antibodies including 5 µl Ki-67-FITC (DAKO A/S, Glostrup, Denmark; approximately 1:30 diluted), 5 µl CD14-PE (RMO52, Beckman Coulter), 3 µl CD64-ECD (22, Beckman Coulter), 5 µl CD13-PE-Cy5.5 (Immu103.44, Beckman Coulter), 5 µl CD117-PE-Cy7 (104D2D1, Beckman Coulter), 5 µl CD34-APC (581, Beckman Coulter), 3 µl CD10-APC-A700 (ALB1, Beckman Coulter), 1 µl CD11b-APC-A750 (Bear1, Beckman Coulter), 5 µl HLA-DR-PB (Immu-357, Beckman Coulter), 2 µl CD45-KO (J.33, Beckman Coulter).

Fifty µl of BM sample (diluted to white blood cell count < 20 × 10^9^/L) was incubated with the respective antibodies for 15 minutes at RT in the dark for extracellular staining. After incubation, cells were washed with 4 mL of PBS and centrifuged for 5 min at 300 × g.

Non-nucleated cell lysis, fixation and permeabilization of cells was performed with the Fix&Perm buffer set (GAS-002; Nordic-MUbio, The Netherlands) according to the manufacturer's protocol for intranuclear staining with Ki-67-FITC (DAKO A/S, Glostrup, Denmark; diluted 1:30). After intranuclear staining, cells were washed in 4 mL PBS and centrifuged for 5 min at 300 × g. Cells were then resuspended in 0.5 mL of PBS. Within 2 hours of immunolabeling, samples were analyzed by flow cytometry.

The Navios™ Flow Cytometer (Beckman Coulter) in combination with the Navios Tetra software (Beckman Coulter) was used for data acquisition. Instrument setup and the daily verification of the optical alignment and fluidics system of the Navios™ Flow Cytometer with Flow-Check™ Pro Fluorospheres (Beckman Coulter) were performed according to standard procedures. Weekly verification of the compensation for each fluorochrome was performed with the Flow-Set™ Pro Fluorospheres (Beckman Coulter).

Ideally, 500,000 relevant events per tube were acquired, while a minimum of 100,000 relevant per tube was deemed acceptable. Nevertheless, a minimum of 100 Ki-67 positive cells per myeloid cell population were measured to ensure accurate quantification of these cells. The number of relevant events was defined as BM cells excluding debris with the FSC-Area vs. SSC-Area plot, but without exclusion of the erythroid cells.

In the case of an insufficient number of events, we opted for analysis of multiple tubes at once with the “Merge and Calculate” function of the Infinicyt 2.0 software package (Cytognos, Salamanca, Spain). A minimum of 1000 events of the cell population of interest were included in the “Merge and Calculate” procedure. Backbone markers were included according to the guidelines of the EuroFlow consortium and comprised FSC, SSC, CD13, CD34, CD45, CD117 and HLA-DR [3]. This allowed proper selection of homogeneous populations of blast cells, erythroid cells, granulocytes and monocytes.

### 2.3 Gating of the different hematopoietic cell populations

**Fig. 1:**
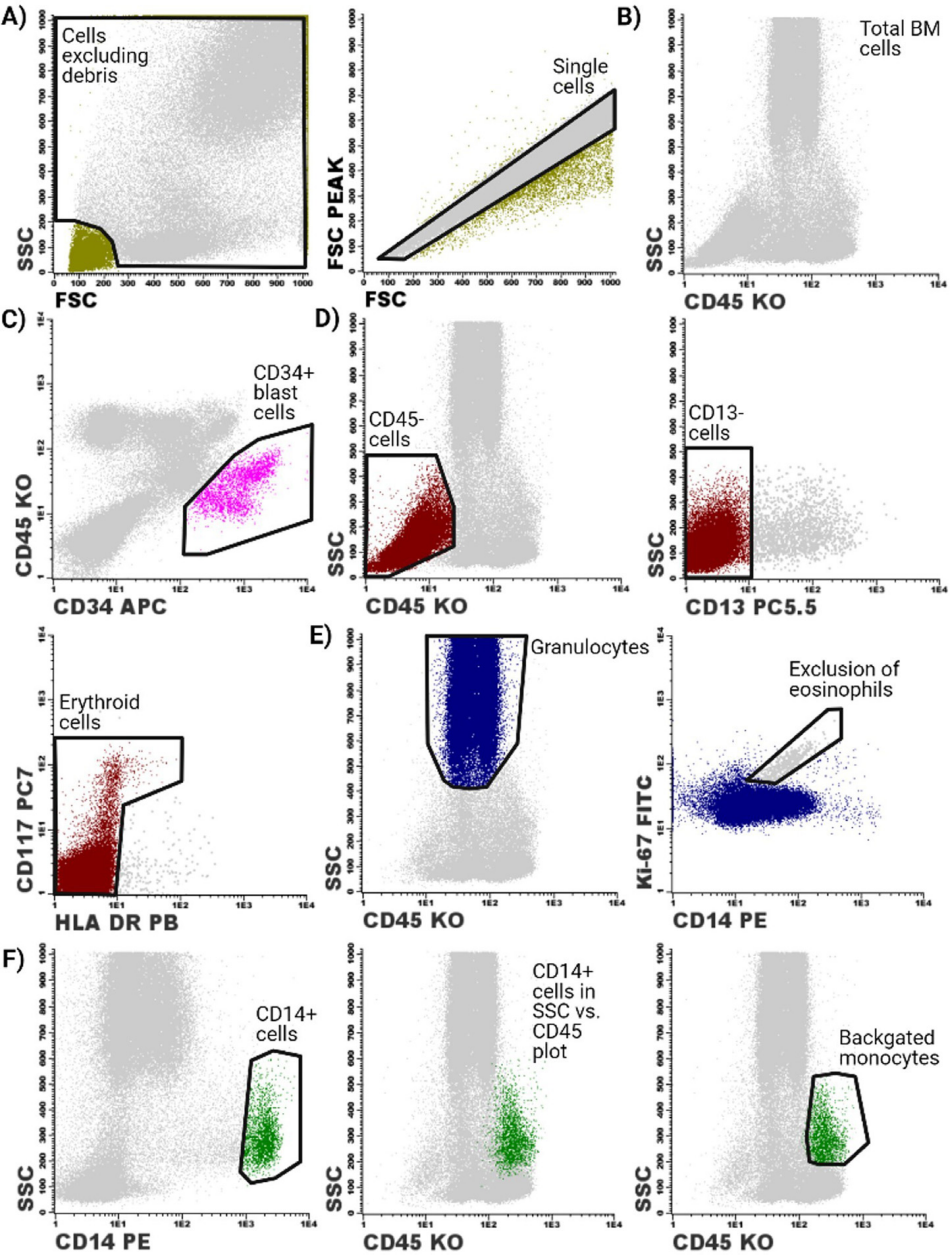
**Gating strategy for determination of the different cell populations in the BM.** A) Debris and doublets were first excluded from the single cells. B) Exclusion of debris and doublets yielded the total BM cell population as shown in the SSC vs. CD45 plot. C) The CD34 positive were gated based on their CD45 dim CD34 positive phenotype. D) CD45 negative CD13 negative cells were then gated, followed by the selection of the erythroid cells based on their characteristic CD117 and HLA-DR expression pattern. E) Granulocytes were gated based on their high SSC and intermediate CD45 expression. Contaminating eosinophils were then excluded from the granulocyte population. F) The total monocyte population was gated based on a backgating procedure. The gating of mature monocytes acted as guidance for gating the total monocyte population in the SSC vs. CD45 plot. Backgating of the total monocyte population was performed by gating the total monocyte population in the SSC vs. CD45 plot, followed by removal of the gate of the mature monocytes in the SSC vs. CD14 plot.

The gating strategy for determination of the Ki-67 proliferation indices of the different cell populations in the BM is illustrated in Fig. 1. Fig. 1A shows that debris and doublets were excluded first. Fig. 1B illustrates that exclusion of debris and doublets yielded the total BM cell population. Subsequently, the CD34 positive blast cells were gated (Fig. 1C). Gating of the erythroid cells as shown in Fig. 1D was adapted from Matarraz et al [4]. CD45 negative cells were gated in the SSC vs. CD45 plot, followed by selection of the CD13 negative cells. Erythroid cells were then selected by their characteristic CD117 and HLA-DR expression pattern. The granulocytes were then selected based on their high SSC and CD45 dim phenotype, followed by exclusion of eosinophils from this population based on the high autofluorescence of the eosinophils (as displayed in Fig. 1E). Fig. 1F shows the gating of mature monocytes in the SSC vs. CD14 plot. As mature monocytes are primarily non-proliferative, we aimed to include immature monocytes by means of a backgating strategy. The gating of mature monocytes acted as guidance for gating the total monocyte population in the SSC vs. CD45 plot. Backgating of the total monocyte population was performed by gating the total monocyte population in the SSC vs. CD45 plot, while removing the gate of the mature monocytes in the SSC vs. CD14 plot.

### 2.4 Determination of the Ki-67 proliferation index by different gating strategies

**Fig. 2:**
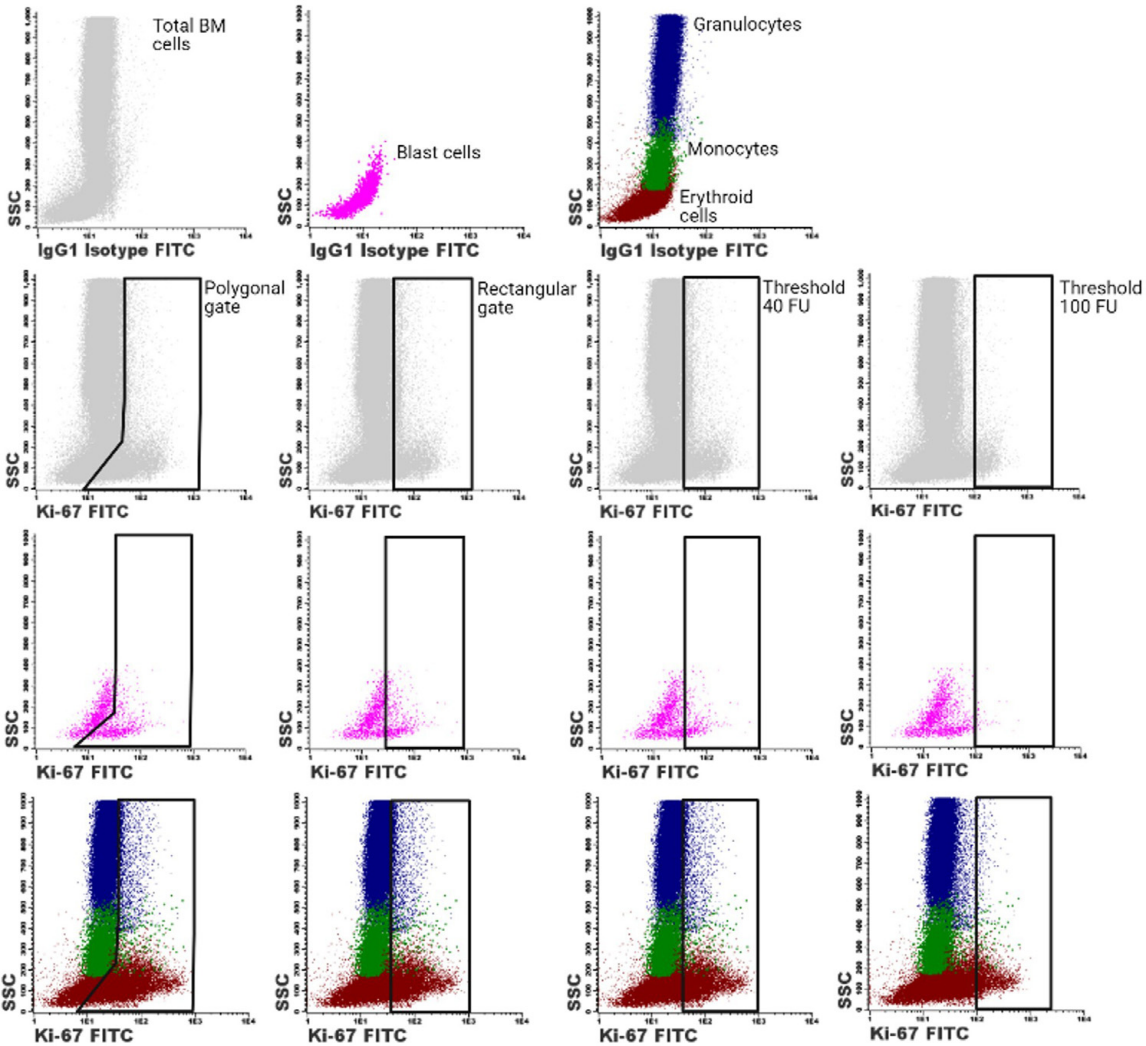
**Different gating procedures for determination of the Ki-67 proliferation index in total BM cells (shown in grey), CD34 positive blast cells (pink), erythroid cells (brown), granulocytes (blue) and monocytes (green).** Ki-67 proliferation indices were quantified by manually placed polygonal and rectangular gates. Alternatively, Ki-67 proliferation indices were quantified by placing the gates based on a predefined threshold based on the fluorescent intensity of the Ki-67 staining (40 FU and 100 FU).

Gating the Ki-67-FITC positive fractions of the different cell populations is a critical step in this assay. Therefore, we investigated whether using manually placed rectangular gates and predefined gating thresholds based on fluorescence intensities produced similar results as compared to placing polygonal gates as described in our previous studies (Fig. 2) [2,5]. Polygonal and rectangular gates were placed according to the IgG1 isotype control-FITC and the Ki-67 negative population as an internal biological control. When using the “Merge and Calculate” function, placement of gates based on the IgG1 isotype control-FITC proved to be most reliable. The “Merge and Calculate” function introduced a low degree of noise signal in the cell populations, which created difficulties for gate placement of the Ki-67-FITC positive fraction based on the internal biological control. The “Merge and Calculate” function had integrated software-based controls to assess whether the introduced degree of noise signal remained in acceptable ranges, which proved to be the case. The Ki-67 proliferation indices of the different myeloid BM cell populations were also determined based on predefined gating thresholds of 40 fluorescent units (FU) and 100 FU. The predefined gating threshold of 40 FU was chosen, since this was the average gating threshold of the manual gating procedure. The threshold of 100 FU was chosen to investigate whether cells that exhibit high levels of Ki-67 expression were of value for diagnostic purposes.

### 2.5 Statistical analysis

The GraphPad Prism 8.0 software package (GraphPad Software, San Diego, USA) was used for statistical analysis. Normality was tested using the Kolmogorov-Smirnov test. Based on the distribution of the data (normal distribution or non-normal distribution) differences in Ki-67 proliferation indices between MDS and non-clonal cytopenic control patients were estimated with the Independent T-test or Mann-Whitney U test, respectively. The different significance levels were defined and are indicated in the figures as *p<0.05, **p<0.01 and ***p<0.001.

**Fig. 3:**
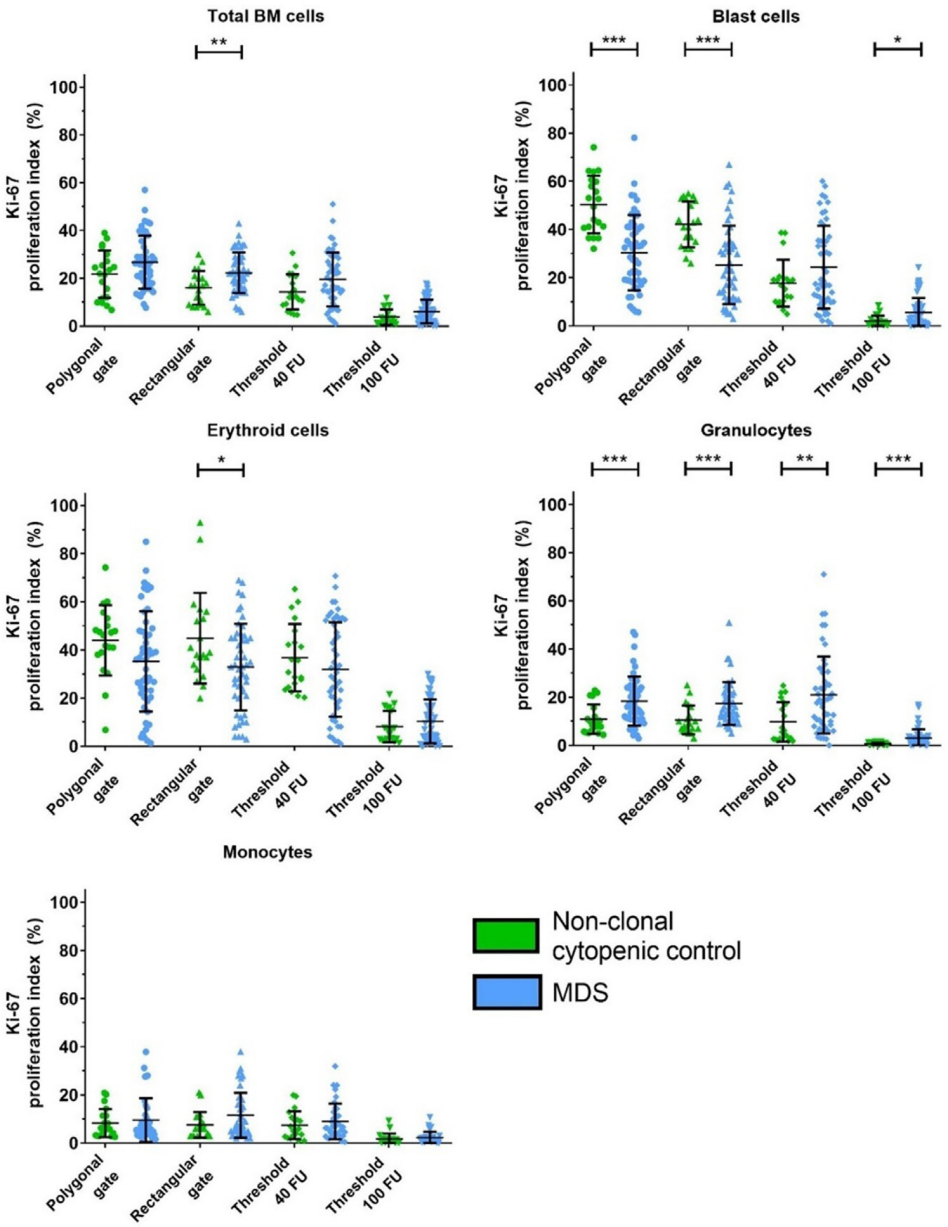
**Influence of the different gating types on the quantification of the Ki-67 proliferation indices of the myeloid BM cell populations.** Horizontal lines depict the mean Ki-67 proliferation index, and the whiskers depict the standard deviation. (Significance levels are indicated as follows: * = p<0.05; ** = p<0.01; *** = p<0.001). Differences between the Ki-67 proliferation index of non-clonal cytopenic patients and that of MDS patients were more pronounced with the use of rectangular gates as compared to the use of polygonal gates. The differences between the Ki-67 proliferation indices of non-clonal cytopenic patients and MDS patients diminished when using predefined gating thresholds of 40 FU and 100 FU. Figure based on the data displayed in the Supplemental Data File.

## Ethics Statements

Data was acquired after informed consent in accordance with the Declaration of Helsinki, while also approval for this study was obtained from the Medical Ethical Committee of the Zuyderland Medical Center and Hogeschool Zuyd (METC Z registration nr. 15-N-201).

## CRediT Author Statement

**Stefan G.C. Mestrum:** Data curation, Formal analysis, Investigation, Methodology, Writing – original draft, Writing – review & editing; **Eline M.P. Cremers:** Investigation, Writing – original draft, Writing – review & editing; **Norbert C.J. de Wit:** Investigation, Writing – original draft, Writing – review & editing; **Roosmarie J.M. Drent:** Formal analysis, Investigation, Methodology, Supervision, Writing – original draft, Writing – review & editing; **F.C.S. Ramaekers:** Conceptualization, Data curation, Investigation, Methodology, Supervision, Writing – original draft, writing – review & editing; **A.H.N. Hopman:** Conceptualization, Data curation, Investigation, Methodology, Supervision, Writing – original draft, Writing – review & editing; **M.P.G. Leers:** Conceptualization, Data curation, Investigation, Methodology, Supervision, Writing – original draft, Writing – review & editing.

## Declaration of Competing Interest

The authors declare that they have no known competing financial interests or personal relationships that could have appeared to influence the work reported in this paper.

The authors declare the following financial interests/personal relationships which may be considered as potential competing interests.

F.C.S.R. is CSO and QA manager at Nordic-MUbio, Susteren, The Netherlands.

## Data Availability

Flow cytometry data for determination of the Ki-67 proliferation index for diagnosis of myelodysplastic syndromes (Original data) (FlowRepository).

## Appendix Supplementary materials

Image, application 1
